# Effects of prenatal mobile phone radiation exposure on MMP9 expression: Implications for inflammation, oxidative stress, and sensory-motor impairment after neonatal hypoxia- ischemia in rats

**DOI:** 10.1016/j.toxrep.2023.10.007

**Published:** 2023-10-13

**Authors:** Samira Khayat, Hamed Fanaei, Narges Lakzaee

**Affiliations:** a Pregnancy Health Research Center, Zahedan University of Medical Sciences, Zahedan, Iran; b Department of Physiology, School of Medicine, Zahedan University of Medical Sciences, Zahedan, Iran

**Keywords:** Cell phone use, Radiation effects, Hypoxia-ischemia, MMP-9, Pregnancy

## Abstract

**Objective:**

Non-ionizing radiofrequency radiation, which finds application in various sectors such as industry, commerce, medicine, and particularly in mobile phone technology, has emerged as a topic of significant concern during pregnancy. The aim of this study was to investigate the effect of cell phone radio-frequency (RF) radiation during pregnancy on the Matrix metalloproteinase 2 (MMP-2) and (MMP-9) 9 expressions after neonatal hypoxia-ischemia (HI) in rats.

**Materials and methods:**

Two groups were formed by randomly assigning female Wistar rats: Group 1 consisted of female rats that were not exposed to RF radiation during pregnancy, while Group 2 comprised female rats that were exposed to RF radiation during pregnancy. After delivery, male offspring were divided into four groups including: (a) SHAM, (b) Exposure (EXP), (c) hypoxia-ischemia (HI), (d) HI/Exposure (HI/EXP). Seven days after HI induction, neurobehavioral tests were performed, and then brain tissue was taken from the skull to measure MMP-2 and MMP-9 expressions, inflammation, oxidative stress, infarct volume and cerebral edema.

**Results:**

MMP-9 mRNA expression in the HI/EXP group was significantly higher than the HI, SHAM and EXP groups. MMP-2 mRNA expression levels in the HI group were significantly higher than Sham and the EXP groups.

TNF-α and Total oxidant capacity (TOC) levels in the HI/EXP group were significantly higher than HI, EXP and SHAM groups. Total antioxidant capacity (TAC) level in the HI/EXP group were significantly lower than HI, EXP and SHAM groups. Cerebral edema and infarct volume in the HI/EXP group were significantly greater than the HI group. Sensory-motor function was significantly weaker in HI/EXP as compared HI group.

**Conclusion:**

Our findings indicate that during pregnancy, exposure to mobile phone RF radiation intensifies damage from HI in rat pups by elevating MMP-9 activity.

## Highlights

• This study explored impact of mobile phone radiation during pregnancy on neonatal hypoxia-ischemia (HI) in rats.
• The exposure group had higher MMP-9 expression, inflammation, oxidative stress, cerebral edema and infarct volume.
• Findings suggest mobile phone exposure during pregnancy amplifies HI-induced damage in rat pups via increased MMP9 activity.

## Graphical Abstract

**Figure:**
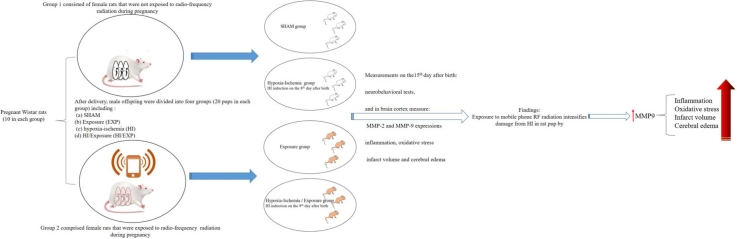

## 1 Introduction

Non-ionizing radiofrequency (RF) radiation, which finds use in various sectors such as industry, commerce, medicine, and particularly in mobile phone technology, has emerged as a topic of significant concern in recent times [1]. Neonatal hypoxia-ischemia (HI) is a destructive condition and the most common cause of death and brain injury in neonates [2], [3].

The long-term disabilities, such as cerebral palsy, mental retardation, cognitive, motor, and sensory disorders, observed in survivors of neonatal HI are the result of significant damage to the developing brain. During HI, there is a disruption of the oxygen and blood supply to the brain, leading to a cascade of events (e.g. inflammation, oxidative stress, and energy failure) that contribute to brain injury [3], [4], [5]. Matrix Metalloproteinases (MMPs) collectively play a crucial role in the pathophysiology of neonatal HI [6], [7]. The dysregulation of MMPs during HI contributes to the progression of brain injury [6], [7]. MMPs, particularly MMP-2 and MMP-9, are involved in the breakdown of the components of the blood-brain barrier (BBB) after neonatal HI [8]. The BBB maintains the integrity of the brain's microenvironment by selectively allowing the passage of certain substances from the bloodstream to the brain [8]. During neonatal HI, an upregulation of MMPs can degrade the tight junction proteins and extracellular matrix surrounding blood vessels, leading to increased BBB permeability [8]. This allows the entry of inflammatory cells and molecules into the brain, further exacerbating the injury [8]. MMPs contribute to the inflammatory response associated with neonatal HI [9]. The release of MMPs, particularly MMP-9, promotes the breakdown of extracellular matrix components and the release of pro-inflammatory cytokines and chemokines. This inflammatory response attracts immune cells to the site of injury, leading to further tissue damage and the production of reactive oxygen species [5], [9].

There is no effective treatment for neonatal HI up to now [2], [10], [11]. The major treatments available are supportive to prevent the spread of injury after HI [2], [11]. However, all neonates with HI experience varying degrees of neurological and behavioral disorders [2], [3]. So, it is important to identify and minimize the factors that aggravate and increase the chance of neonatal HI. Research on the effects of mobile waves on matrix metalloproteinases (MMPs) is an ongoing area of investigation. A growing frame of evidence suggests that exposure to mobile waves, specifically radiofrequency electromagnetic fields (RF-EMF), may influence MMP activity and expression [12], [13]. These changes in MMPs can impact various physiological processes, including tissue remodeling, inflammation, and cell migration [12], [13]. However, it is worth noting that the specific mechanisms and potential health implications of these effects are still being studied, and further research is needed to fully understand the relationship between mobile waves and MMPs.

Examining the effects of mobile RF on MMPs in neonatal HI is crucial for understanding potential mechanisms underlying brain injury in affected newborns. Perturbations in MMPs activity due to mobile RF exposure during HI may exacerbate brain damage and contribute to long-term neurological outcomes. Understanding this relationship can aid in identifying preventive measures or interventions to minimize the impact of mobile phone waves on MMP-mediated pathophysiological processes. Further research in this area is essential for optimizing care and improving outcomes for neonates affected by HI. Therefore, this study aimed to investigate the impact of prenatal exposure to mobile RF radiation on the expression of MMP-2 and MMP-9 subsequent to neonatal HI.

## 2 Materials and methods

### 2.1 Animals

The Faculty of Medical Ethics Committee for animal Research of Zahedan University of Medical Sciences, approved this study (ethical code: IR.ZAUMS.REC.1398.179). This study utilized female Wistar rats weighing between 200 and 220 g, which were sourced from the Laboratory Animal Research Center of Zahedan University of Medical Sciences. The animals were acclimated for one week prior to the experiment, during which they were housed in controlled environmental conditions with unrestricted access to water and food. These conditions included a 12:12 h day/night cycle and a temperature maintained at 22 ± 2 C.

### 2.2 Experimental design

In order to induce pregnancy in rats, a cage was set up containing two female Wistar rats and one male Wistar rat. Subsequently, the animals' vaginas were examined for sperm every morning [14]. Rats that tested positive were separated and kept in another cage [4], [14]. Next, twenty pregnant rats were separated into two groups (10 in each group): First group: Rats were not exposed to mobile phone RF radiation (12 h daily) during pregnancy: the pups of Sham (n = 20) and the Hypoxia-ischemic (HI) groups (n = 20) were selected from the male offspring of these rats. HI was induced in the pups by occluding the right Common Carotid Artery and exposing them to 8% oxygen for 90 min [4].

Second group: Rats were exposed to mobile phone RF radiation during pregnancy: Dams of this group exposed to RF radiation (900 MHz) from the first day of gestation until the end of study. The pups of exposure (EXP) (n = 20) and hypoxia-ischemia/exposure (HI/EXP) (n = 20) groups were selected from male offspring of these animals.

### 2.3 Exposure to mobile phone RF radiation

The second group of female rats was exposed to 900 MHz mobile phone RF radiation generated by a mobile-wave frequency generator (Mobile Frequency Simulator device, Bionic Mobin Co) with an output power of 2 watts/kg. The exposure started from the first day of pregnancy and continued until the end of the study, with a duration of 12 h per day [15]. The power density was measured using the SPECTRAN HF-6060 V4 device equipped with MCS Spectrum Analyzer Software (Aaronia, Germany). The measurement revealed a peak power density of 0.045 µW/cm2 at a distance of 20 cm from the antenna. The first group rats maintained in the same condition in mobile-wave frequency generator without exposure to RF radiation. On postnatal day 1, male offspring were divided into four groups (20 in each group):1- Sham group (SHAM): In this group, surgery was performed but the right Common Carotid Artery (CCA) was not occluded and hypoxia was not induced. 2- Exposure (EXP) group: The right CCA of the pups were not occluded and they did not experienced hypoxia (pups of this group were selected from dams that exposed to RF radiation during pregnancy). 3- Hypoxia-ischemic group (HI): In this group right CCA was occluded and hypoxia was induced (exposed to 8% oxygen for 90 min [4]). 4- HI/EXP group: The right CCA of pups was occluded, and then exposed to 8% oxygen for 90 min (pups of this group were selected from dams that exposed to RF radiation during pregnancy).

### 2.4 Neonatal hypoxia-ischemia induction

For neonatal HI induction, the Rice-Vannucci method was used [4], [16]. On the 8th day after birth, the pups were anesthetized with ketamine (80 mg/kg) and xylazin (10 mg/kg). Then, the right CCA of pups were occluded by a nylon silk suture 6–0. One hour after recovery, pups were exposed to 8% oxygen for 90 min [4]. On postnatal 15th day neurobehavioral tests were done [17], then offsprings brain tissue was taken from their skull to measure MMP2, MMP9, TNF-α, oxidative stress, cerebral edema and infarct volume.

### 2.5 Real-time quantitative PCR (RT-qPCR)

For the measurement of MMP2 and MMP9 mRNA expression, RT-qPCR was conducted in this study. Total RNA was extracted from the ischemic cerebral cortex hemisphere using TRIzol reagent (Invitrogen, Shanghai, China). Subsequently, the extracted mRNA was subjected to reverse transcription to generate cDNA, which was performed using the cDNA Reverse Transcription kit (Qiagen, USA) following the manufacturer's instructions. Amplification of the cDNAs was achieved using the miScript SYBR Green PCR Kit (Qiagen) as per the manufacturer's guidelines. The primer sequences used for MMP9 were as follows: forward primer 5′- AGGTGCCTCGGATGGTTATCG −3′ and reverse primer 5′- TGCTTGCCCAGGAAGACGAA −3′. The primer sequences for MMP2 were: forward primer 5′- AAAGGAGGGCTGCATTGTGAA −3′ and reverse primer 5′- CTGGGGAAGGACGTGAAGAGG −3′. To determine the relative expression levels of MMP2 and MMP9, the 2^−ΔΔCt^ method was employed, with GAPDH serving as the internal reference gene.

## 3 Neurobehavioral tests

### 3.1 Cliff avoidance test

The cliff avoidance test assesses the coordination between sensory input and motor output in rat pups [4]. To conduct this test, a platform measuring 30 cm by 30 cm was utilized [4]. On the 15th day after birth, the pups were positioned with their chest at the edge of the platform, supported by all four limbs [4]. Subsequently, the duration of their response, whether rotational movement or retreat from the platform's edge, was recorded. In cases where the pup fell off or remained immobile for 60 s, a duration of 60 s was recorded [4], [18].

### 3.2 Negative geotaxis test

The negative geotaxis test is utilized to evaluate the sensory-motor function in rat pups. On the 15th day after birth, the rat pups were positioned on an inclined surface with a slope of 30 degrees. The pups' heads were positioned facing downward along the slope of the surface. The duration it took for the pups to turn upwards (180 degrees) was then recorded. The maximum recorded time for this test was 90 s [4].

### 3.3 Evaluation of cerebral edema and infarction volume

Once the neurobehavioral tests were finished, the rat pups were anesthetized and their brains were extracted from their skulls. The brain tissues were then sliced using a coronal brain matrix, resulting in 2 mm thick sections. The ischemic area was exposed by immersing the slices in a 2% solution of Triphenyl Tetrazolium Chloride (TTC) [4]. A scanner (Scanjet, Hewlett-Packard, USA) captured an image of the slice. The Image J software (NIH) was used to measure the size of the ischemic region in the images. The following formula was used to calculate the ischemic area volume[4], [19]:

$$\mathrm{Volume of ischemic region}=(\frac{\mathrm{volume of contralateral hemisphere}-\mathrm{the volume of the nonischemic region of the ipsilateral hemisphere}}{\mathrm{volume of contralateral hemisphere}})\times 100$$

To measure cerebral edema, following formula was used [4], [20]:

$$\mathrm{Edema}=\frac{\left(\mathrm{volume of ipsilateral hemisphere}-\mathrm{volume of contralateral hemisphere}\right)}{\mathrm{volume of contralateral hemisphere}}$$

### 3.4 Measuring the level of TNF-α and oxidative stress in pups brain

On the 15th day after birth, the puppies were given anesthesia and euthanized. The brain tissues were extracted and placed in a cold phosphate-buffered saline solution. Afterwards, the samples were homogenized and centrifuged at 1370g for 15 min, resulting in the collection of supernatants. Finally, TNF-α, Total antioxidant capacity (TAC), and total oxidant capacity (TOC) were measured using specific kits (all kits obtained from Zellbio, Germany).

### 3.5 Statistical analysis

The data analysis was performed using GraphPad Prism Ver. 8. Statistical analysis was conducted using one-way ANOVA followed by Bonferroni tests. The data are presented as mean ± standard error of the mean (SEM). A significance level of P < 0.05 was considered as indicating a significant difference.

## 4 Result

### 4.1 MMP-2 and MMP-9 gene expressions in pup brain tissue

**Fig. 1:**
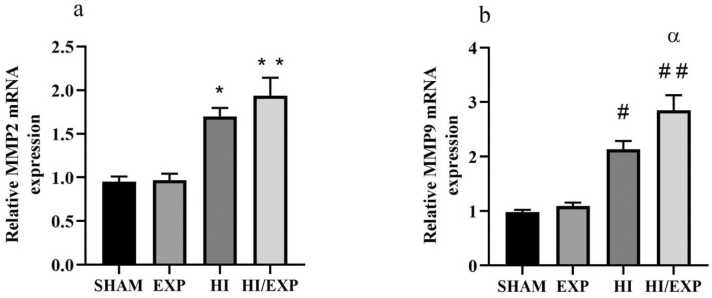
Gene expression of (a) MMP2 and (b) MMP9 in brain cortex of SHAM, EXP, HI and HI/EXP groups. (a) * p < 0.01, HI vs. SHAM and EXP; ** p < 0.0001 HI/EXP vs. SHAM and EXP, (b) # p < 0.001, HI vs. SHAM and EXP; ## p < 0.0001 HI/EXP vs. SHAM and EXP, α p < 0.05, HI/EXP vs. HI (mean ± SEM, n = 8). MMP2 (Matrix Metallopeptidase 2), MMP9 (Matrix metallopeptidase 9), EXP (exposure), HI (hypoxia-ischemia), HI/EXP (hypoxia-ischemia/exposure).

As demonstrated in Fig. 1a, relative MMP-2 mRNA expression level in the HI/EXP group was higher than HI group, but this difference was not significant. The relative MMP-2 mRNA expression level in the HI group was significantly higher as compared to the Sham and the EXP (both p < 0.01) groups. Fig. 1b displays that the relative MMP-9 mRNA expression in the HI/EXP group was significantly higher than the HI (p < 0.05), SHAM (p < 0.0001) and EXP (p < 0.0001) groups. Relative MMP-9 mRNA expression in HI group was significantly higher than SHAM and EXP groups (both p < 0.001).

### 4.2 TNF-α levels and oxidative stress status in brain tissue

**Fig. 2:**
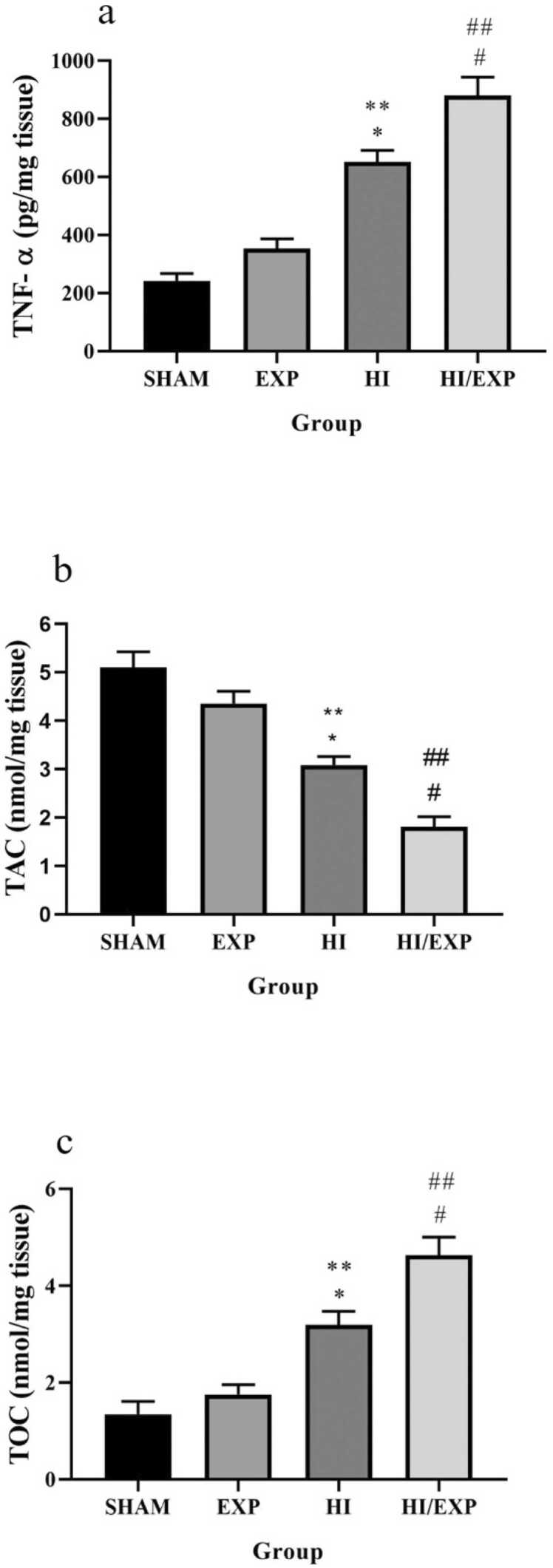
TNF-α, TAC and TOC levels in SHAM, EXP, HI and HI/EXP groups. (a) * p < 0.0001, HI vs. SHAM; ** p < 0.001, HI vs. EXP; # p < 0.0001 HI/EXP vs. SHAM and EXP; ## p < 0.01, HI/EXP vs. HI. (b) * P < 0.0001, HI vs. SHAM; ** P < 0.01, HI vs. EXP; # P < 0.0001 HI/EXP vs. SHAM and EXP; ## P < 0.01 HI/EXP vs. HI (c) * p < 0.001, HI vs. SHAM; ** p < 0.01, HI vs. EXP; # p < 0.0001, HI/EXP vs. SHAM and EXP; ## P < 0.01, HI/EXP vs. HI. TNF-α (Tumour necrosis factor α), TAC (Total antioxidant capacity), TOC (Total oxidant capacity), EXP (exposure), HI (hypoxia-ischemia), HI/EXP (hypoxia-ischemia/exposure).

The mean of TNF-α level in HI/EXP group was significantly more than HI (p < 0.01), Sham (p < 0.0001) and EXP groups (p < 0.0001) (Fig. 2a). TNF-α level in the brain tissue of the HI group was significantly higher than sham (p < 0.0001) and EXP (p < 0.001) groups. As shown in Fig. 2b, TAC level in HI/EXP group was significantly lower than HI (p < 0.01), Sham (p < 0.0001) and EXP (p < 0.0001) groups. Mean of TAC level in the HI group was significantly lower than in the sham (p < 0.0001) and EXP (p < 0. 01) groups. Moreover, the mean of TAC level in the EXP group was lower than sham group, but this difference was not significant.

TOC level in HI/EXP group was significantly higher than HI (p < 0.01), Sham (p < 0.0001), EXP (p < 0.0001) groups (Fig. 4). The mean of TOC level in HI group was significantly higher than Sham (p < 0.001) and EXP (p < 0.01) groups.

**Fig. 3:**
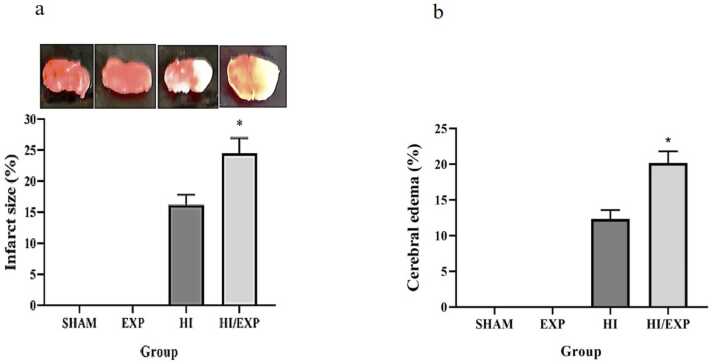
Infarct size and cerebral edema in SHAM, EXP, HI and HI/EXP groups. (a) * p = 0.0182, HI/EXP vs. HI, (b) * p = 0.0037, HI/EXP vs. HI. EXP (exposure), HI (hypoxia-ischemia), HI/EXP (hypoxia-ischemia/exposure).

**Fig. 4:**
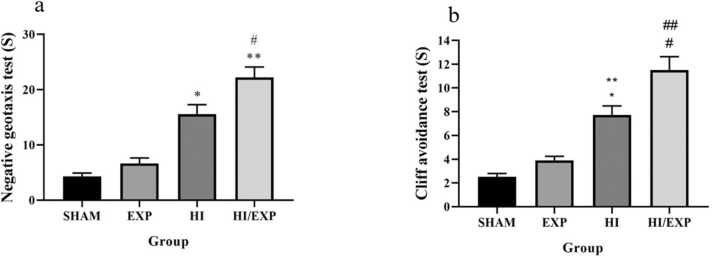
Negative geotaxis and Cliff avoidance tests in SHAM, EXP, HI and HI/EXP groups. * p < 0.0001, HI vs. SHAM and EXP; ** p < 0.0001, HI/EXP vs. SHAM and EXP; # p < 0.01, HI/EXP vs. HI, (b) * p < 0.0001, HI vs. SHAM; ** p < 0.01, HI vs. EXP; # p < 0.0001, HI/EXP vs. SHAM and EXP; ## p < 0.01, HI/EXP vs. HI. EXP (exposure), HI (hypoxia-ischemia), HI/EXP (hypoxia-ischemia/exposure).

### 4.3 Infarct size and cerebral edema

The percentage of infarct volume in HI/EXP group was significantly greater than HI group (p = 0.0182) (Fig. 3a). Cerebral edema in the HI/EXP group was significantly higher than the HI group (p = 0.0037) (Fig. 3b).

### 4.4 Negative geotaxis and Cliff avoidance tests

Latency of negative geotaxis test in the HI group was significantly higher than sham (p < 0.0001) and EXP (p < 0.0001) groups. Latency in HI/EXP group was significantly longer than HI (p < 0.01), EXP (p < 0.0001) and sham (p < 0.0001) groups (Fig. 4a). As shown in Fig. 4b, latency of cliff avoidance test in the HI was significantly higher than sham (p < 0.0001) and EXP (p < 0.01) groups. Latency in the HI/EXP group was significantly longer than HI (p < 0.01), Sham (p < 0.0001) and EXP (p < 0.0001) groups (Fig. 4b).

## 5 Discussion

In the physiological conditions during the postnatal period, MMP9 plays important roles in the neural network development, myelination and plasticity, especially in the cerebral cortex [21]. Therefore, the abnormal change of MMP-9 activity during the critical period of the newborn's development can lead to irreparable sensory, motor and psychological damages [6], [21].

The findings of our study showed that exposure to mobile phone RF during pregnancy has been linked to heightened activity of MMP-2 and MMP-9 and an increase in neonatal HI damage. This suggests a potential association between mobile wave exposure and the modulation of MMPs expression, which plays a role in tissue remodeling and inflammatory responses. The impact of increased MMP9 activity in the context of neonatal HI can exacerbate brain injury and potentially affect long-term outcomes[6].

Further research is needed to fully understand and establish the mechanisms underlying this relationship and determine appropriate preventive measures. In line with our study, Azimipour et. al (2020) showed Exposure to cell phone radiation affects the growth and maturation rate of murine ovarian follicles by altering the expression of MMP-2 and MMP-9 genes [12]. In another study, Friedman et. al. (2007) discovered that mobile phone electromagnetic irradiation triggers the activation of plasma membrane NADH oxidase, which leads to the generation of reactive oxygen species (ROS) [13]. These ROS, in turn, directly stimulate the activity of matrix metalloproteinases (MMPs) [13]. In addition, the results of our study showed that exposure to mobile phone RF radiation during pregnancy increases the level of inflammation and oxidative stress, brain edema and the volume of the infarcted area, which leads to the aggravation of brain damage after neonatal HI. In our study, the greater increase in inflammation in the group exposed to mobile phone RF radiation could be due to the greater increase in MMP9 expression.

Research has indicated that MMP9 may have a beneficial impact on nerve regeneration by promoting axon growth and axon pad finding after injury in rats [21]. However, it is worth noting that the positive effects are often counteracted by the inflammation triggered by its activity [21].

The increase in MMP9 activity after HI plays a role in promoting inflammation [6], [9]. MMP9 is an enzyme involved in the breakdown of extracellular matrix components, but it also has additional functions beyond tissue remodeling [6], [9], [21]. Following neonatal HI, the increased expression and activity of MMP9 can lead to the breakdown of components of the Blood-Brain Barrier (BBB) [6], [9]. This disruption compromises the integrity of the BBB, allowing inflammatory cells and molecules to infiltrate the brain tissue more easily, leading to an increased inflammatory response [6], [9].

Our findings indicate that the higher elevation of oxidative stress observed in the group exposed to mobile waves may be attributed to the more significant upregulation of MMP9 expression. Increased MMP9 after HI can contribute to elevated oxidative stress levels [9], [22]. This is primarily due to the role of MMP9 in promoting the breakdown of the extracellular matrix (ECM) and disrupting the BBB [9], [22], [23].

During HI, there is a decrease in oxygen and nutrient supply to the brain, leading to cellular energy depletion and subsequent cell death [4], [9]. This process triggers an inflammatory response, including the activation of MMP9 [6], [9]. MMP9 is a protease that degrades various components of the ECM, such as collagen and laminin [24]. By breaking down these structural proteins, MMP9 contributes to BBB disruption, allowing immune cells and other molecules to infiltrate into the brain parenchyma[9].

The infiltration of immune cells further exacerbates oxidative stress by releasing ROS and pro-inflammatory cytokines [9]. ROS are highly reactive molecules that can damage cellular components, including lipids, proteins, and DNA [4], [9]. This leads to an imbalance between ROS production and antioxidant defense mechanisms, resulting in increased oxidative stress. Moreover, MMP9 can directly induce oxidative stress by activating pro-oxidant enzymes like NADPH oxidase [25]. NADPH oxidase generates superoxide radicals, which are potent ROS [25]. These radicals can further contribute to oxidative damage in HI [25].

Overall, increased MMP9 levels during neonatal HI lead to BBB disruption and immune cell infiltration, resulting in increased production of ROS and pro-inflammatory cytokines [9], [25]. This cascade of events ultimately leads to enhanced oxidative stress within the brain.

Our findings suggest that the larger infarct volume and cerebral edema observed in the group exposed to mobile waves can be attributed to the more pronounced increase in MMP9 expression. The increase in MMP9 following cerebral ischemia has been associated with an expansion of the ischemic area and the development of cerebral edema [26]. Elevated MMP9 levels contribute to these effects through various mechanisms [26]. Increased MMP9 activity can lead to the degradation of components of the BBB, compromising its integrity [26]. This allows the extravasation of fluid, blood cells, and inflammatory factors into the surrounding brain tissue, exacerbating both ischemic injury and cerebral edema [26]. MMP9 facilitates the breakdown of extracellular matrix proteins, contributing to tissue remodeling processes [26]. However, it is important to note that the proper regulation of MMP-9 is crucial for various aspects of healthy brain development and plasticity in postnatal period. Dysregulation of MMP-9 levels and activity has been increasingly linked to neurodevelopmental and psychiatric disorders characterized by abnormal brain development [21].

Excessive MMP9 activity can disrupt the balance between matrix degradation and repair, exacerbating tissue damage and promoting the expansion of the ischemic area [26]. MMP9 plays a role in the inflammatory response following cerebral ischemia [26]. Increased MMP9 expression leads to the release of pro-inflammatory cytokines, chemokines, and immune cell recruitment, which promote tissue damage, edema formation, and expansion of the ischemic area [26]. MMP9 can influence the regulation of blood vessels in the brain [26]. Excessive MMP9 levels impair the ability of blood vessels to constrict or dilate properly, leading to altered blood flow regulation and contributing to the development of cerebral edema and expansion of the ischemic area [26].

In summary, the increase in MMP9 following cerebral ischemia promotes BBB disruption, extracellular matrix remodeling, inflammation, impaired cerebrovascular regulation, and oxidative stress, which collectively contribute to the expansion of the ischemic area and the development of cerebral edema.

From a therapeutic perspective, previous studies have demonstrated the capability of certain drugs, such as diazepam and minocycline, to inhibit MMP9 activity [21]. Considering this information, it is recommended to explore the effects of these drugs in future studies. Investigating the impact of diazepam and minocycline on MMP9 inhibition could provide valuable insights into potential therapeutic interventions for mitigating the consequences of mobile wave-induced brain injury after neonatal HI.

## 6 Conclusion

In conclusion, the findings of this study suggest that exposure to mobile waves during pregnancy can have detrimental effects on neonatal health, particularly in cases of neonatal hypoxia-ischemia. The group exposed to mobile waves during pregnancy showed increased inflammation, oxidative stress, and worsened infarct volume and cerebral edema compared to the group that did not have exposure to mobile waves. These effects were attributed to the increased expression of MMP9, a protein associated with tissue damage and inflammation. These results highlight the potential risks of mobile wave exposure during pregnancy and emphasize the importance of further research in this area to better understand its long-term consequences on neonatal health, warranting the need for deeper understanding and targeted interventions to improve outcomes for this vulnerable population.

## Ethics approval statement

The study was approved by Ethics Committee of Zahedan University of Medical Sciences (ethical code: IR.ZAUMS.REC. 1398.179).

## Author contributions

Hamed Fanaei and Samira Khayat designed the study. Narges Lakzaee, Hamed Fanaei, and Samira Khayat carried out the experiments. Hamed Fanaei and Samira Khayat analyzed the data. Hamed Fanaei and Samira Khayat wrote the manuscript.

## Funding

Financial support for the study was conducted by the Office of Vice-President for Research and Information Technology of 10.13039/501100004847Zahedan University of Medical Sciences (code number: 9476).

## Declaration of Competing Interest

The authors declare that they have no known competing financial interests or personal relationships that could have appeared to influence the work reported in this paper.

## Data Availability

Data will be made available on request.

## References

[1] Deshmukh P.S., Megha K., Banerjee B.D., Ahmed R.S., Chandna S., Abegaonkar M.P. (2013). Detection of low level microwave radiation induced deoxyribonucleic acid damage Vis-à-vis genotoxicity in brain of fischer rats. Toxicol. Int..

[2] Bano S., Chaudhary V., Garga U.C. (2017). Neonatal hypoxic-ischemic encephalopathy: a radiological review. J. Pedia Neurosci..

[3] Millar L.J., Shi L., Hoerder-Suabedissen A., Molnár Z. (2017). Neonatal hypoxia ischaemia: mechanisms, models, and therapeutic challenges. Front. Cell Neurosci..

[4] Bornavard M., Fanaei H., Mirshekar M.A., Farajian Mashhadi F., Atashpanjeh A. (2020). Morphine consumption during pregnancy exacerbates neonatal hypoxia-ischemia injury in rats. Int. J. Dev. Neurosci.: Off. J. Int. Soc. Dev. Neurosci..

[5] Li B., Concepcion K., Meng X., Zhang L. (2017). Brain-immune interactions in perinatal hypoxic-ischemic brain injury. Prog. Neurobiol..

[6] Salah M.M., Abdelmawla M.A., Eid S.R., Hasanin R.M., Mostafa E.A., Abdelhameed M.W. (2019). Role of matrix metalloproteinase-9 in neonatal hypoxic-ischemic encephalopathy. Open Access Maced. J. Med. Sci..

[7] Leonardo C.C., Pennypacker K.R. (2009). Neuroinflammation and MMPs: potential therapeutic targets in neonatal hypoxic-ischemic injury. J. Neuroinflamm..

[8] Zhang W., Zhang H., Mu H., Zhu W., Jiang X., Hu X. (2016). Omega-3 polyunsaturated fatty acids mitigate blood-brain barrier disruption after hypoxic-ischemic brain injury. Neurobiol. Dis..

[9] Okazaki K., Nishida A., Kimura H., Buonocore G., Bracci R., Weindling M. (2016). Neonatology: A Practical Approach to Neonatal Diseases.

[10] Sudan M., Birks L.E., Aurrekoetxea J.J., Ferrero A., Gallastegi M., Guxens M. (2018). Maternal cell phone use during pregnancy and child cognition at age 5 years in 3 birth cohorts. Environ. Int..

[11] Lai M.-C., Yang S.-N. (2011). Perinatal hypoxic-ischemic encephalopathy. J. Biomed. Biotechnol..

[12] Azimipour F., Zavareh S., Lashkarbolouki T. (2019). The effect of radiation emitted by cell phone on the gelatinolytic activity of matrix metalloproteinase-2 and -9 of mouse pre-antral follicles during in vitro culture. Cell J..

[13] Friedman J., Kraus S., Hauptman Y., Schiff Y., Seger R. (2007). Mechanism of short-term ERK activation by electromagnetic fields at mobile phone frequencies. Biochem. J..

[14] Rezaei S., Bakhshani N.M., Fanaei H., Trofimova I. (2021). Opium effect in pregnancy on the dynamics of maternal behavior: testing a neurochemical model. Neuropsychobiology.

[15] Houston B.J., Nixon B., McEwan K.E., Martin J.H., King B.V., Aitken R.J. (2019). Whole-body exposures to radiofrequency-electromagnetic energy can cause DNA damage in mouse spermatozoa via an oxidative mechanism. Sci. Rep..

[16] Rice J.E., Vannucci R.C., Brierley J.B. (1981). The influence of immaturity on hypoxic-ischemic brain damage in the rat. Ann. Neurol..

[17] Azadbakht Z., Fanaei H., Karajibani M., Montazerifar F. (2023). Effect of type 1 diabetes during pregnancy and lactation on neonatal hypoxia-ischemia injury and apoptotic gene expression. Int. J. Dev. Neurosci..

[18] Lai P.C., Huang Y.T., Wu C.C., Lai C.J., Wang P.J., Chiu T.H. (2011). Ceftriaxone attenuates hypoxic-ischemic brain injury in neonatal rats. J. Biomed. Sci..

[19] Chelluboina B., Warhekar A., Dillard M., Klopfenstein J.D., Pinson D.M., Wang D.Z. (2015). Post-transcriptional inactivation of matrix metalloproteinase-12 after focal cerebral ischemia attenuates brain damage. Sci. Rep..

[20] Kollmar R., Frietsch T., Georgiadis D., Schabitz W.R., Waschke K.F., Kuschinsky W. (2002). Early effects of acid-base management during hypothermia on cerebral infarct volume edema and cerebral blood flow in acute focal cerebral ischemia in rats. Anesthesiology.

[21] Reinhard S.M., Razak K., Ethell I.M. (2015). A delicate balance: role of MMP-9 in brain development and pathophysiology of neurodevelopmental disorders. Front. Cell Neurosci..

[22] Kelly P.J., Morrow J.D., Ning M., Koroshetz W., Lo E.H., Terry E. (2008). Oxidative stress and matrix metalloproteinase-9 in acute ischemic stroke: the biomarker evaluation for antioxidant therapies in stroke (BEAT-Stroke) study. Stroke.

[23] Chen Q., Jin M., Yang F., Zhu J., Xiao Q., Zhang L. (2013). Matrix metalloproteinases: inflammatory regulators of cell behaviors in vascular formation and remodeling. Mediat. Inflamm..

[24] Yabluchanskiy A., Ma Y., Iyer R.P., Hall M.E., Lindsey M.L. (2013). Matrix metalloproteinase-9: many shades of function in cardiovascular disease. Physiology.

[25] Tang X., Zhong W., Tu Q., Ding B. (2014). NADPH oxidase mediates the expression of MMP-9 in cerebral tissue after ischemia-reperfusion damage. Neurol. Res..

[26] Dong X., Song Y.N., Liu W.G., Guo X.L. (2009). Mmp-9 a potential target for cerebral ischemic treatment. Curr. Neuropharmacol..

